# Design of Security Paper with Selective Frequency Reflection Characteristics

**DOI:** 10.3390/s18072263

**Published:** 2018-07-13

**Authors:** Sang-Hwa Lee, Min-Sik Kim, Jong-Kyu Kim, Ic-Pyo Hong

**Affiliations:** 1 Department of Information and Communication Engineering, Kongju National University, Gongju 31080, Korea; leesh0915@smail.kongju.ac.kr; 2 National Security Research Institute, Daejeon 34188, Korea; kms76@nsr.re.kr (M.-S.K.); contai@nsr.re.kr (J.-K.K.)

**Keywords:** security paper, frequency selective structure, document leakage detection, angle of incidence, four-legged element, screen printing method

## Abstract

In this research, a security paper based on frequency selective structure technologies was designed and fabricated using selective wave reflection characteristics to prevent the offline leakage of confidential documents. Document leakage detection systems using security papers detect security papers using transceiving antenna gates. For the application of such systems, the structure must be designed with excellent reflection performance and stability at the angle of incidence. For this purpose, a loop and patch-type frequency selective structure based on a four-legged element structure was designed to have X-band frequency reflection characteristics. This design was based on optimized variables and was realized through the screen printing method using silver ink on A4 paper. It was verified that both the design and simulation results matched well. To verify its actual applicability, a detector module operable at 10 GHz was manufactured to observe both the security paper detection range in relation to distance with a signal strength of −10 dBm and the detection area in relation to the number of times that the security paper had been folded.

## 1. Introduction

In today’s rapidly changing information-oriented society, much effort has been devoted to securing national competitiveness through the development of unique technologies. However, as advanced technologies become connected to national core technologies, the risk of technology leakage is growing. The outflow of core technologies causes enormous losses by damaging the national economy and hindering industrial development. In particular, in the case of the national defense industry, the outflow of core technologies constitutes dangerous criminal acts that threaten the security of the nation. Moreover, leaked industrial secrets are irrecoverable and difficult to compensate. Therefore, it is necessary to actively work to develop systems related to the protection of domestic industrial technologies [1,2].

In most cases, industrial secrets are directly or indirectly leaked with the help of insiders through various routes and media. Leakage cases carried out through online routes such as e-mail and messenger services can be tracked and used for criminal investigation after leaks have occurred. To prevent the occurrence of such leaks in advance, electronic document systems optimized for effective information management and security have been introduced through the digitization of information. However, the risk of hacking or data replication has not been totally mitigated. Therefore, documents with special purposes, such as design drawings or documents having legal effects, are still produced and kept in the form of paper documents.

Since the outflow of paper documents through offline routes such as fax and mail allow criminal acts to be stopped before confidential information is leaked, offline routes are the focus of efforts to prevent confidential leakage through in-advance detection. Thus, the development of a system to prevent offline data leakage is required. To prevent data leakage in offline environments, papers with security functions, such as uncopiable papers, copy-prevention papers, and electronic surveillance papers are being used [3,4].

Among these, the electronic article surveillance (EAS) system equips papers with electromagnetic induction sensors so that the outflow of papers can be detected at antenna gates located at the exits when printed documents are taken outside of the security area due to theft or leakage, causing an alarm to sound. These systems are divided into the electromagnetic (EM) type, which uses papers with magnetic induction materials, and the radio Frequency (RF) type, which uses papers equipped with radio frequency identification (RFID) tags. The EM type uses tags made of soft magnetic materials inside the paper, allowing the detection of harmonics generated by magnetic field changes caused when the paper passes through the alternating magnetic field created by the detector [5]. In 2002 [6], a study was made to fabricate security paper by applying magnetic strip structure to paper. The proposed technology can produce a security paper with a built-in magnetic sensor for each paper, but the magnetic strip structure operates at several kHz frequency and has a short recognition range. In addition, since the magnetic field is formed differently depending on the shape and direction of the magnetic strip, it is difficult to stably detect the paper. The RF type uses RFID tags with specific LC values created by conductive materials inside the paper, making it resonate at specific frequencies and creating strong electrical signals that can be detected after it comes into contact with a reader. It also features fast recognition speed and long-range detection. However, the general RFID structure uses an Intergrated Circuit (IC) chip to implement the capacitance component and the ID, and it is difficult to construct the security paper by applying it to each paper sheet in terms of structural and economic aspects [7]. To improve these disadvantages, a chipless-RFID was designed and printed on A4 paper. The RFID was printed on the reader, and the tag and resonance could be confirmed at the point of alignment. However, it can be recognized only when it is very close. Through previous research, RFID technology is an effective technology for managing books, files, and document bundles, but it is a technology that is difficult to apply to a single document [8].

In this research, to develop a microwave-based electronic surveillance paper, a security paper was created based on frequency selective surface (FSS) production technologies using cost-effective printed electronic technology. FSSs have a structure that acts as a space filter due to the periodic arrangement of conductive materials on a dielectric substrate, and they are used for indoor frequency control, radomes, and wave absorbers [9,10,11]. The traditional method of FSS circuit fabrication uses a printed circuit board (PCB) on an epoxy substrate. Recently, there has been a study on FSS which is manufactured by a printing method using conductive ink on a flexible substrate such as film or paper. There are a lot of film-based studies because it is easy to form a conductive pattern using a printing method on a film and a high-resolution FSS can be produced according to a printing technique when a film is used as a substrate to fabricate an FSS [12,13,14]. A study on FSS based on paper has been carried out on FSS with Wi-Fi band cut-off characteristics produced by a printing method using paper as a substrate in 2015 [15]. In 2016, there was a study on FSS with General System Mobile (GSM) band-stopping characteristics by forming a conductive pattern by adhering a copper film on paper [16]. In the same year, there was a study on reconstructed FSS based on coated paper based on printing method [17]. In the case of fabricating FSS by a printing method using a paper as a substrate, due to the nature of paper as a fibrous material, the conductive ink is not printed or printed with a very high surface resistance due to the property of absorbing ink. In addition, most studies using film and paper based flexible substrates are aimed at frequency blocking to improve the propagation environment of the indoor environment and focus on improving the frequency blocking performance and blocking the multi-band frequency. In this paper, we focus on the frequency reflection characteristics of the security paper using the proposed FSS, and it is necessary to design the unit structure to improve the reflection performance and to maintain the uniform surface condition even if there is a conductive pattern inside the security paper. It is necessary to manufacture the product in consideration of the internal applicability, easiness of manufacture, and economic problems. In addition, to solve the problem that paper absorbs ink to weaken conductivity, use of ink through viscosity control is required.

**Figure 1:**
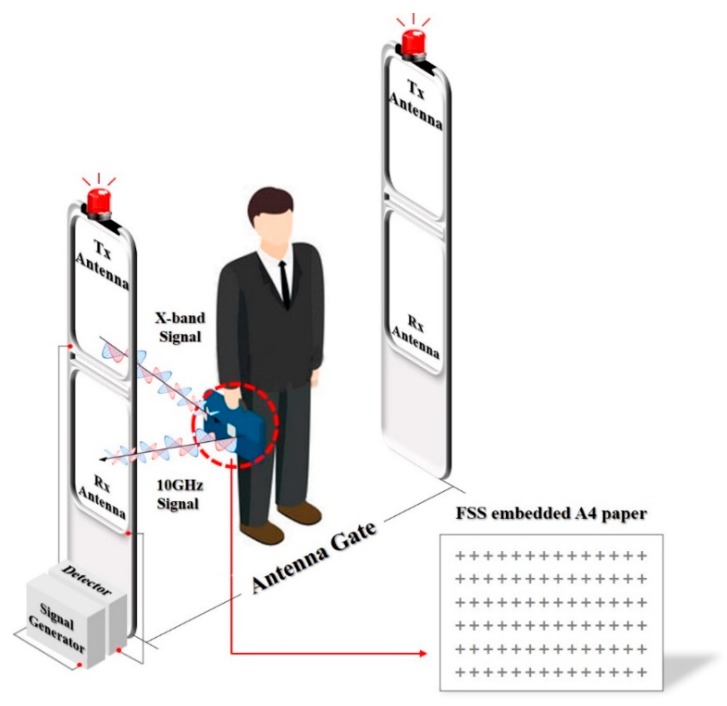
Operation principle of security paper detecting system.

In this research, to develop security paper to prevent the leakage of industrial technologies in offline environments, an A4-paper screen printing method using silver ink was applied to create electromagnetic-wave security paper that operates in the X-band. The frequency reflection properties of this security paper were measured, and its applicability was verified through testing using detectors. Figure 1 shows a security paper detection system proposed and designed in this paper by applying a detector and a signal generator to an EAS system with antenna gates [18].

## 2. Design and Fabrication of the Security Paper

### 2.1. Design of Frequency Selective Structure

**Figure 2:**
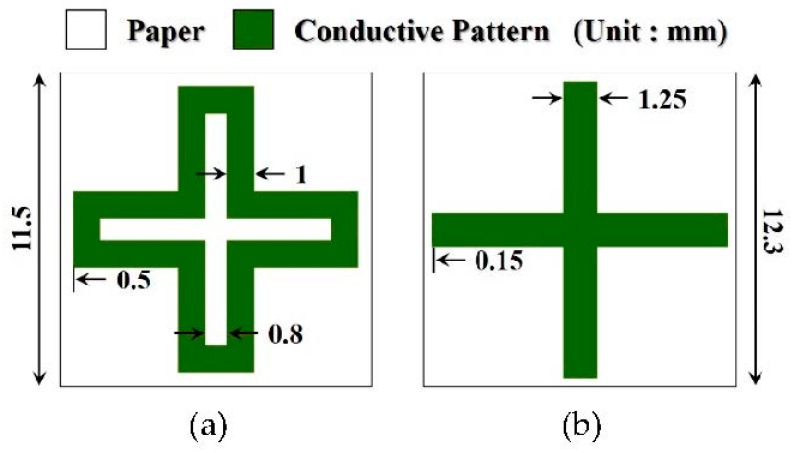
Proposed frequency selective surface unit cell: (**a**) Loop type; (**b**) Patch type.

**Figure 3:**
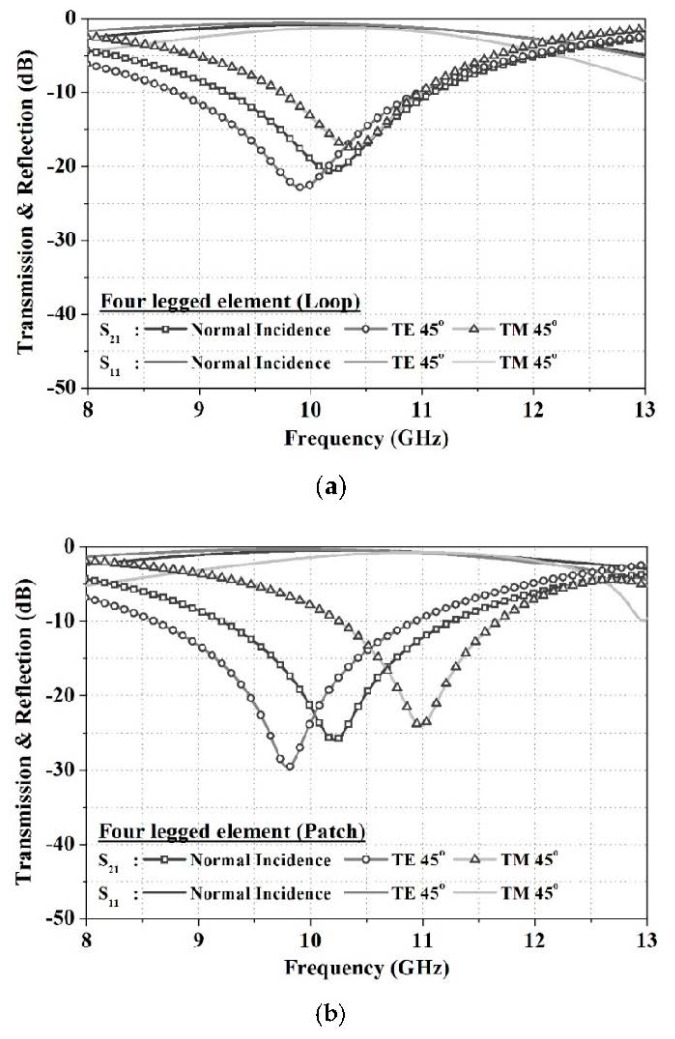
Simulated transmission and reflection results of frequency selective surface (FSS): (**a**) Loop type; (**b**) Patch type.

The location and status of papers that pass through antenna gates are unpredictable. To enable their detection, patterns must be designed that have excellent frequency reflection characteristics and are stable to the angle of incidence and for the polarization of incident waves. The frequency selective structure is designed with a loop structure to secure the incidence-angle stability through miniaturization based on a four-legged element structure, which is known to have narrowband characteristics [19]. The line width and size of the units in the structure are related proportionally. Therefore, the narrower the line width, the smaller the unit cell design becomes, and the higher the stability against the incident angle becomes. The surface resistance decreases as the line width of the conductor becomes wider, and the thickness becomes thicker. In this paper, to reduce the size of the FSS unit structure, we designed the FSS structure with a line width of 1 mm by increasing the thickness through multiple printing while reducing the line width. The incidence-angle stability was verified to be 2.5% (260 MHz), and the bandwidth was verified to be 18.2% (1.86 GHz) based on a vertical incidence permeation loss of −10 dB. Although the loop structure is stable to the angle of incidence, the patch structure was designed to improve the transmission performance. The incidence-angle stability of the patch structure was verified to be 7.2% (740 MHz), and its bandwidth was verified to be 19.6% (2 GHz) based on a transmission loss of −10 dB at the vertical incidence. When the patch structure was used, the transmission performance improved to −5.38 dB, but the stability for an angle of incidence decreased by 4.7% in comparison to the loop structure based on a vertical incidence. The proposed FSS unit structure is shown in Figure 2. A simulation was conducted using the commercial electromagnetic analysis software HFSS, and the results are shown in Figure 3. After designing a unit structure in a vacuum box, which is a free space environment, the FSS analysis method using HFSS software, we set the master/slave boundary method as an infinite periodic structural analysis and interpreted it as a floquet port as an input signal method. The proposed structure was assumed to have a surface resistance of 0.4 Ω/sq, which satisfies a performance of more than –20 dB transmission loss. The actual silver ink has a very low sheet resistance value of about 0.02 Ω/sq. However, paper made from fibrous materials absorbs conductive ink and, therefore, increases the surface resistance, which should be taken into account when simulating. In addition, a paper (εr = 2.8, tan δ = 0.02) with a thickness of 0.1 mm was used as the dielectric substrate.

### 2.2. Fabrication of Security Paper

**Figure 4:**
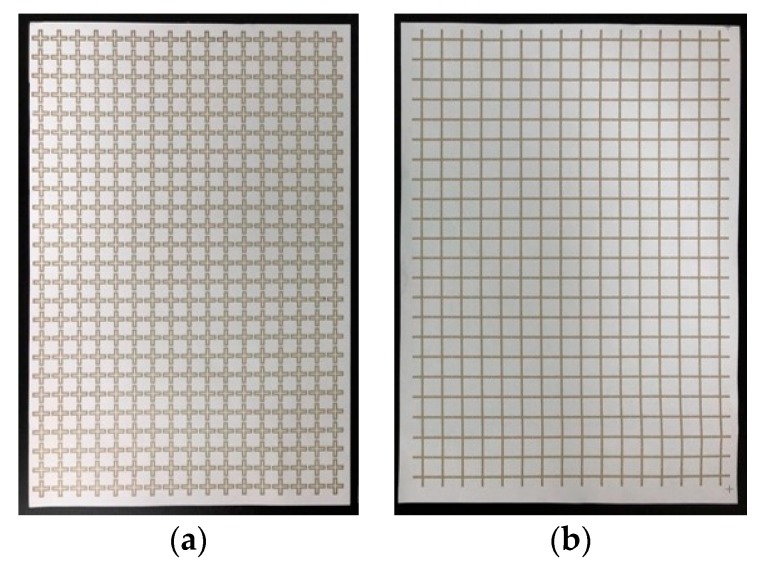
Fabrication FSS on paper: (**a**) Loop type; (**b**) Patch type.

The proposed FSS was fabricated by screen printing on an A4 paper using silver ink with a surface resistance of 0.02 Ω/sq. During printing, it was verified that the paper, which was made of fiber, was not conductive because the low-viscosity conductive ink permeated the paper. To solve this problem, a high-viscosity silver ink of 28,000 cPs was used at 20 °C. Here, 1 cPs is the viscosity of water at 20 °C. A screen printing technique optimized for the use of high-viscosity ink was used. After printing, post-processing including heat, UV-ray, and nitrogen treatment, was carried out to increase the ink’s preservability or durability. Further, to improve the surface resistance of the fabricated security paper, it was heat cured for 20 min at 150 °C. The fabricated FSS is shown in Figure 4. To fit the size of an A4 paper, the loop structure has 425 unit structures in a 25 × 17 array, and the patch structure has 368 unit structures in a 23 × 16 array.

## 3. Security Paper Validation

### 3.1. Measurement of the Frequency Characteristics of Security Paper

**Figure 5:**
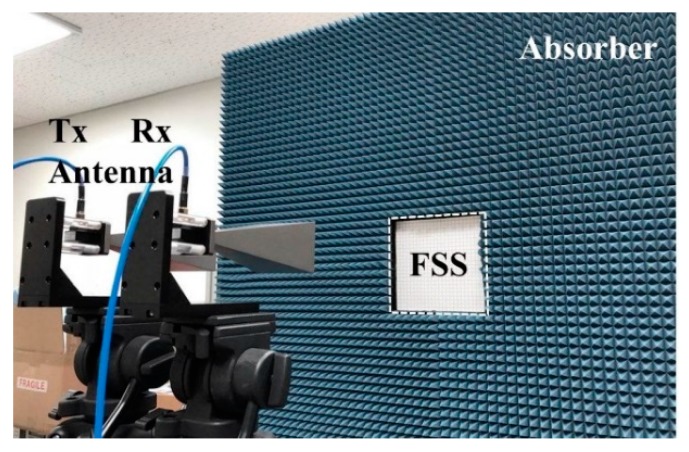
Frequency reflection characteristic measurement setup.

**Figure 6:**
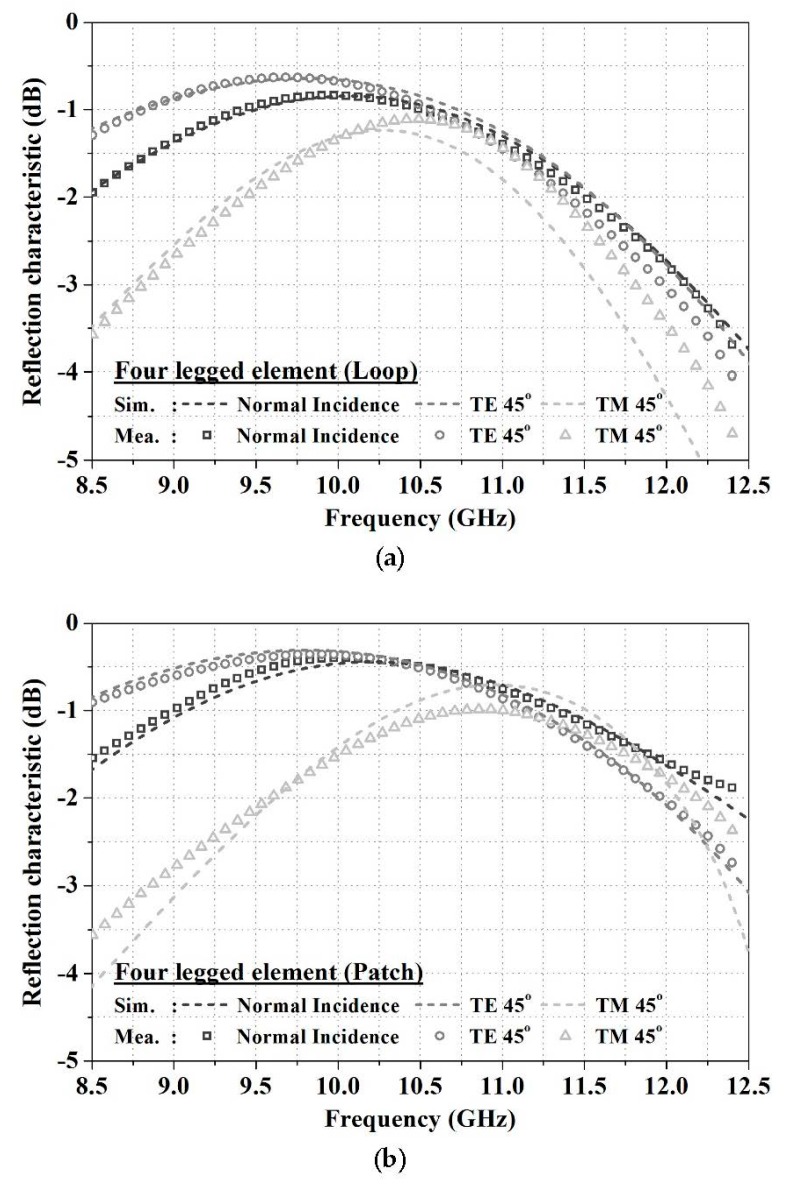
Measurement results of frequency reflection characteristic: (**a**) Loop type; (**b**) Patch type.

The frequency reflection characteristics of the fabricated security paper were measured in the free-space measurement environment shown in Figure 5, using a horn antenna with a band of 8.2 to 12.4 GHz, and a network analyzer. The angle of incidence of the fabricated FSS was measured to be 0° and 45° according to the Transverse Electric (TE)/Transverse Magnetic (TM) polarization of incident waves. As shown in Figure 6, a comparison of simulation and measurement results revealed that the maximum resonant frequency deviations of the loop structure and the patch structure were 1.7% (180 MHz) and 0.5% (60 MHz), respectively, at a TM-polarized angle of incidence of 45°, showing that the measurement results were similar to simulation results. The incidence angle stability of the loop structure was verified to be 5.1% (515 MHz), and that of the patch structure was 8.7% (870 MHz), which means that the loop structure is advantageous for a more stable performance at a TM-polarized incidence angle of 45°.

### 3.2. Measurement of the Security Paper Using a Detector

**Figure 7:**
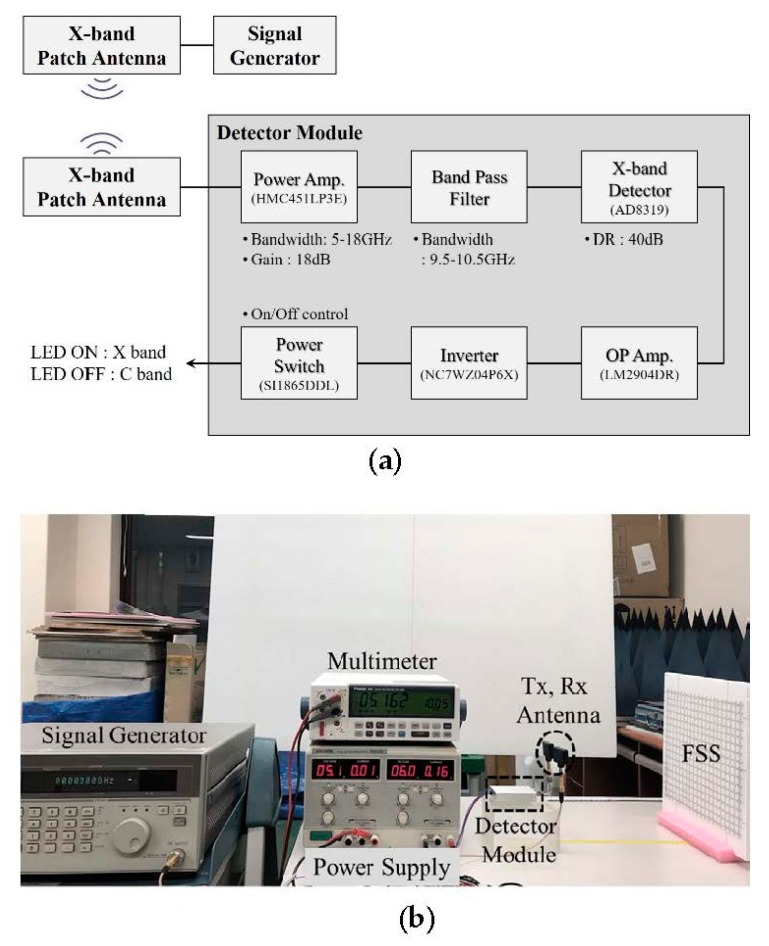
Detector measurement setup: (**a**) block diagram; (**b**) measurement setup.

To create a system in which an alarm sounds when the security paper passes through an antenna gate, a detection system with a detector module equipped with a patch antenna and an LED was created, as shown in Figure 7, and its FSS detection area was observed. As shown in Figure 7, the detection system is composed of a transceiving patch antenna, detector module, network analyzer, signal generator, power supply, and multimeter.

**Figure 8:**
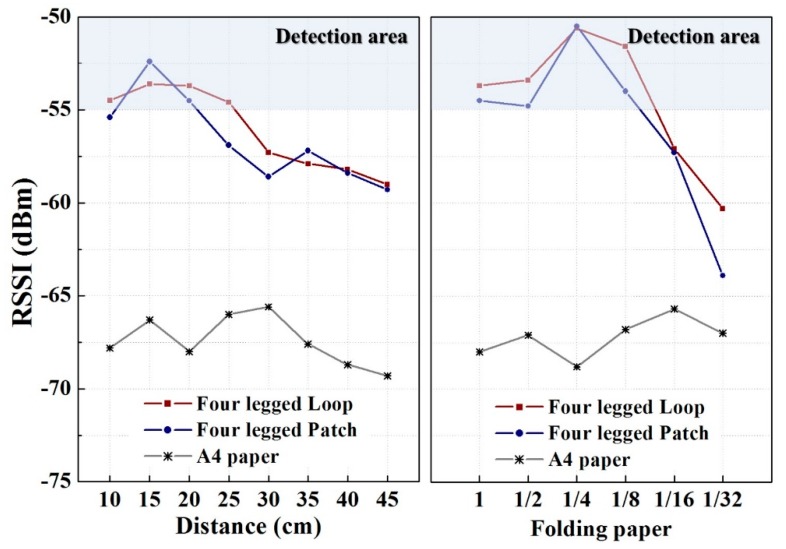
Received signal strength indicators (RSSI) measurement results.

The transmitting antenna receives signals from the signal generator and sends them to the FSS. Then, the receiving antenna receives the reflected signals from the FSS and verifies the detection area through the LED lights of the detector module. The signal generator applies a signal strength of −10 dBm to the transmitting antenna, and the detector module receives power of 158 mA from the power supply at 6 V, and a power of 150 mA from the LED at 5 V. To clearly observe the LED lights, a multimeter was connected to the LED terminal to verify the output of 5 V. To verify the detection area according to the distance of the security paper and to prevent leakage of folded paper with the detector module system, the detection area was observed according to the number of times the paper was folded. The security paper was folded in half five times for the test. The paper size and number of unit structures in relation to the number of folds are tabulated in Table 1. After the detection area was measured by using the detector module, the received signal strength indicators (RSSI) were observed. The measurement results are shown in Figure 8. The loop structure was detectable at distances ranging from 8 to 26 cm, and the patch structure was detectable at distances ranging from 7 to 24 cm. The A4 paper was undetectable when it was smaller than 1/8 its normal size, i.e., when it had been folded more than three times.

## 4. Conclusions

In this research, a security paper was designed and fabricated using frequency selective surface techniques to prevent the outflow of industrial technologies in offline environments. To detect paper passing randomly through antenna gates, a four-legged element structure was designed as a loop and a patch structure to realize a paper with excellent reflection performance and stability to the angle of incidence. Considering the characteristics of security papers, a screen printing technique was used to create the security paper using silver ink on A4 paper. Measurements of its frequency reflection characteristics demonstrated that the fabricated FSS displayed similar results to those obtained by simulation, and the security paper was detectable using the detection system within an average distance of 25 cm. In the future, security paper equipped with a frequency selective structure will be created using laminating technologies to prevent damage of the frequency selective structure exposed on the paper surface.

## Figures and Tables

**Table 1:** Number of unit cell for fold count.

Folding		Number of Unit Cell	
Count	Size (mm)	Loop	Patch
0	A4 (297 × 210)	25 × 17	23 × 16
1	1/2 (210 × 148)	17 × 12	16 × 11
2	1/4 (148 × 105)	12 × 8	11 × 8
3	1/8 (105 × 74)	8 × 6	8 × 5
4	1/16 (74 × 52)	6 × 4	5 × 4
5	1/32 (52 × 37)	4 × 3	4 × 2
